# The Effectiveness of Affective Compared to Neutral Working Memory Training in University Students with Test Anxiety

**DOI:** 10.1016/j.brat.2021.103974

**Published:** 2021-09-24

**Authors:** Savannah Minihan, Zobair Samimi, Susanne Schweizer

**Affiliations:** 1 University of New South Wales, School of Psychology, Developmental Affective Science Lab; 2 International University of Chabahar, Department of Educational Science, Chabahar, Iran; 3 University of Cambridge, Department of Psychology, Developmental Cognitive Neuroscience Group

**Keywords:** Test anxiety, working memory training, cognitive control, emotion regulation

## Abstract

**Background:**

Test anxiety (TA), defined as the emotional, physiological, and behavioural responses surrounding situations involving formal evaluation of performance, is a relatively common occurrence, and, when present, can be a disruptive factor in students’ academic careers. Research indicates that working memory, in particular, affective working memory, is impaired in individuals with TA. The current study therefore explored whether training the application of working memory in affective contexts could reduce TA and associated cognitive and affective impairments.

**Method:**

60 Iranian university students (50% female; 19-22 years) with TA symptoms were randomized to receive 20 sessions of affective working memory training (aWMT), neutral working memory training (nWMT) or to a no-training control group. Prior and immediately after training, all participants completed measures of TA, working memory, cognitive control, and emotion regulation.

**Results:**

Compared to the control group, both the aWMT and the nWMT groups demonstrated improved cognitive and affective functioning from pre- to post-training. However, the reduction in TA symptoms and improvement in emotion regulation was greater in the aWMT group compared to the nWMT group.

**Conclusion:**

aWMT may be an effective means of not only reducing TA, but also enhancing cognitive and affective functioning. These preliminary findings are promising given the potential for free and easy dissemination of aWMT in schools and online settings, including low- and middle-income countries.

Testing has become an integral part of most education systems (Agrawal, 2004). Performance on these tests is thought to reflect student achievement and dictates individuals’ progression throughout their schooling years and beyond to tertiary education access. Yet, there are some students who are so afflicted by anxiety as a result of the testing experience, that they are unable to perform to their full potential (Rothman, 2004). Test anxiety (TA), defined as the emotional, physiological, and behavioural responses surrounding situations involving formal evaluation of performance (Zeidner, 1998), is a relatively common occurrence (Von Der Embse et al., 2013). In studies of university student populations, it has been estimated that 20-40% of students suffer from functionally impairing TA (Gerwing et al., 2015; Naveh-Benjamin et al., 1997; Zeidner, 1998), with research demonstrating negative associations between TA and academic performance (Cassady & Johnson, 2002; Chapell et al., 2005; Naveh-Benjamin et al., 1997; Zeidner, 1998; for a review see von der Embse et al., 2018). TA has also been shown to predict changes in subjective well-being over time (Steinmayr et al., 2016). Given the relatively high prevalence of and negative consequences associated with TA, the development of *scalable*, translational interventions is imperative.

Within the literature, there is consensus that TA comprises two dimensions: worry, which constitutes cognitive concerns and ruminations about performance; and emotionality, which is manifested in physiological responses, such as dizziness or nausea, experienced during evaluative situations (Brown et al., 2011; Cassady & Johnson, 2002; Liebert & Morris, 2016). Research suggests that working memory (WM; a limited capacity cognitive storage system required to maintain and manipulate information (Baddeley, 2003)) may play an important role in TA, particularly the cognitive component. For example, Owens et al. (2012) found that, in a group of typically developing 12- to 13-year-olds, WM capacity mediated the link between worry (the cognitive component of TA) and academic performance. Similarly, in a sample of 11-year-olds, Ng and Lee (2015) observed a direct and detrimental effect of trait TA on WM.

WM capacity is an important individual-difference variable that has been shown to account for a significant proportion of variance in general intellectual ability (Conway et al., 2003; Engle, 2002), as well as being central to the processing of goal-relevant information in the face of goal-irrelevant distraction (Barrett et al., 2004). WM capacity also appears to play an important role in mental health outcomes. Numerous studies have shown impaired WM in depression (Davidovich et al., 2016; Demeyer et al., 2012; Joormann et al., 2011), anxiety (Amir & Bomyea, 2011; Ladouceur et al., 2005), and post-traumatic stress disorder (Schweizer & Dalgleish, 2011, 2016). In mental health disorders, the ability to control the access and inhibition of negative material to and from WM appears especially impaired (de Voogd et al., 2016).

A recent meta-analysis suggested that it may be *affective* WM capacity, in particular, that is impaired in individuals suffering from mental health difficulties (Schweizer et al., 2019). Results showed that, in psychologically healthy individuals, affective information had a negligible impact on WM performance, whereas in those suffering from mental health difficulties, the presence of affective material led to impaired WM performance. Day-to-day cognition, particularly in those with mental health difficulties, typically requires the updating, inhibition, and manipulation of affectively-laden rather than affectively-neutral information in WM. For example, a young person afflicted with TA who is attempting to complete a university entrance exam might simultaneously struggle to set aside intrusive and distressing thoughts about failing the exam.

Few studies have explored affective WM capacity in individuals with TA. However, one recent study provides support for the postulation of impaired affective WM capacity in such individuals. Shi et al. (2014) adapted a modified reading span task from Schweizer & Dalgleish (2011) designed to assess affective WM capacity, by making stimuli specific to TA. Participants high and low in TA were required to perform simple tasks with neutral material (remembering lists of letters) over short intervals while simultaneously dealing with emotionally-laden intrusive thoughts and feelings (processing sentences describing dysfunctional test-related thoughts) relative to neutral facts about the world. Shi and colleagues observed a particular difficulty employing WM in test-related contexts in high TA participants compared to low TA participants. That is, participants’ ability to remember letter lists in the context of test-related sentences, relative to neutral sentences, was poorer in high TA individuals. Training affective WM capacity, then, may be a means of ameliorating TA.

Recent years have seen a proliferation of research exploring the impact of WM training on various outcomes, including mental health symptomatology. The aim of traditional forms of WM training, which incorporates neutral stimuli, is to increase WM capacity and for these improvements to be transferable to real-world contexts (du Toit et al., 2020). However, a series of meta-analytic reviews concluded that WM training does not appear to lead to significant far transfer effects (Au et al., 2015; Melby-Lervåg et al., 2016; Morrison & Chein, 2011). That is, the effectiveness of WM training does not extend beyond the process (i.e., WM) trained. Findings from studies of the impact of neutral WM training (nWMT) on affective processes and clinical outcomes have similarly been mixed (for a review see Motter et al., 2016). For example, Hadwin & Richards (2016) observed reduced anxiety symptoms, increased inhibitory control, and reduced anxiety-related attentional biases in adolescents with elevated anxiety symptoms following nWMT. These effects were comparable to the effects the authors found in a comparison group that underwent Cognitive Behaviour Therapy. Similarly, nWMT, compared to control training, led to increased attentional control and reduced anxiety in high trait anxious individuals (Sari et al., 2016). Conversely, in individuals with elevated rumination (Onraedt & Koster, 2014) and individuals with depression or anxiety (Wanmaker et al., 2015), nWMT did not produce any differential effects on rumination, depression, or anxiety, compared to control training. Given the evidence reviewed suggesting that symptoms of anxiety and depression are associated with affective WM difficulties, training the application of WM in affective contexts may lead to more reliable benefits when targeting affective symptoms, such as TA symptoms.

In support of this notion, Schweizer et al. (2011) demonstrated that both aWMT and nWMT led to transfer gains on another WM task, however, only aWMT training produced transferable gains to improved affective control. Compared to control training, aWMT also led to improved regulation of emotional distress and altered recruitment in the brain regions underlying the successful control of affective information and states (Pan et al., 2020; Schweizer et al., 2013). Beneficial effects of aWMT in clinical populations have also been observed (du Toit et al., 2020; Krause-Utz et al., 2020; Schweizer et al., 2017; however see de Voogd et al., 2016). aWMT then appears to be a promising avenue for improving affective control and symptoms of emotional disorders, such as TA.

Given the promising findings surrounding aWMT and the affective WM deficits observed in individuals with TA, the current study aimed to investigate the effectiveness of aWMT compared to nWMT and a passive control group on TA and related cognitive and affective functions (i.e., WM, cognitive control and emotion regulation). The sample included university students with high TA from a middle-income country. Running the study in a middle-income country demonstrates the potential for disseminating these types of trainings globally at very little cost, in settings where educational attainment is a key determinant of well-being (Graetz et al., 2020).

The aWMT paradigm used for training was adapted from Schweizer et al. (2017) and required participants to update and maintain visual and auditory information in working memory. In this adapted version of the task, the stimuli were replaced to reflect the TA-specific concerns of individuals high in TA. That is, the generic negative facial expressions and words were replaced with TA-related images, e.g., an exam classroom, and TA-related words, e.g., “exam”. The nWMT was identical but included only neutral information (i.e., words and images unrelated to testing such as the word “water” and an image of a mountain). The nWMT group was included to compare the relative effectiveness of training WM in affective versus neutral contexts on improving cognitive and affective functioning, whereas the waitlist control group was included to control for changes due to development and any test-retest effects.

The design allowed us to explore the following hypotheses. First, it was hypothesised that individuals with high TA would be able to train on the aWMT and nWMT paradigms. That is, performance on their respective training tasks at the end of training would be better compared to performance at the start of training (H1: *training hypothesis*). It was also hypothesised that following training, the training groups would show greater gains compared to the control group in WM and cognitive control, as measured on an untrained digit span and GoNogo task, respectively (H2: *cognitive transfer hypothesis*). Finally, it was predicted that only individuals who received aWMT, but not the nWMT or control groups, would show improvements in affective functioning, in the form of enhanced emotion regulation, and reduced levels of TA symptoms (H3: *affective transfer hypothesis*).

## Method

### Participants

Sixty university students (30 female; *M_age_* (*SD*) = 20.5 (1.08), age range = 19 to 22) with TA were recruited from Azarbaijan Shahid Madani University in Tabriz, Iran. The sample size was selected to have 95% power^1^ at α = 0.05 to detect an effect of training on cognitive and affective transfer measures, based on previous effect sizes from studies using the same training paradigm (Schweizer et al., 2013, 2017). In order to participate in the study, participants needed to score greater than 50 on the Test Anxiety Inventory (Spielberger, 1980). A cut-off score of 50 was chosen to ensure that participants had a greater than average level of TA. Spielberger (1980) reported males and females to have an average score of 38.48 (*SD* = 12.43) and 42.79 (*SD* = 13.70) on the Test Anxiety Inventory, respectively. All participants additionally completed a clinical interview (conducted by ZS), in which their TA symptoms were further assessed. The clinical interview contained open-ended questions on the basis of DSM-IV criteria and lasted approximately 10 minutes. Participants were asked about the feelings and anxiety symptoms they experience during exams, the conditions under which they experience such test-related feelings and anxiety, and whether they are currently receiving any intervention for their TA. No participants reported receiving any interventions. The main purpose of the clinical interview was to confirm that TA was present amongst all participants.

Following inclusion, participants were randomly allocated to the aWMT group (*n* = 20, 10 female), the nWMT group (*n* = 20, 10 female) or the control group (*n* = 20, 10 female), stratified by gender.

### Training Tasks

#### Affective Working Memory

**Figure 1:**
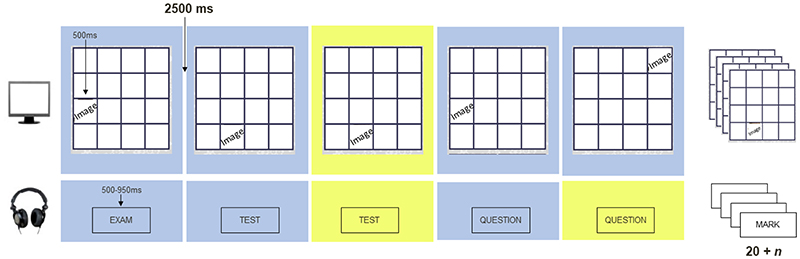
Depiction of Affective Working Memory Training. This figure depicts a block of Affective Working Memory Training, where *n* = 1. Trials depicted with a yellow background represent target stimuli. Participants respond with a button press to indicate whether either or both stimuli match the stimuli presented *n*-trials back any time from the onset of the stimuli to 2500ms, after which the next word-image pairing appears. For an image match to occur, the image must be presented in the same location as the image *n* trials back, while for an auditory match to occur, the word has to be an exact match to the word presented *n*-trials back.

The aWMT (adapted version of the task used by Schweizer et al., 2017), depicted in Figure 1, comprised an affective dual *n*-back task consisting of a series of trials each of which involved simultaneous presentation of an image (500ms) on a 4 x 4 grid on a laptop screen and a word (500-950ms) over headphones. Participants pressed a button to indicate whether either or both stimuli matched the stimuli presented *n*-trials back any time from the onset of the stimuli to 2500ms, after which the next word-image pairing appeared. For an auditory match to occur, the word had to be an exact match to the word presented *n*-trials back. In contrast, for a match to occur in the visuospatial modality, an image had to appear in the same location as the image *n*-trials back. That is, the content shown in the image was task-irrelevant and had to be inhibited. Participants were required to only attend, maintain and respond to the location of the images that were presented. The training then trained both engagement (auditory modality) and disengagement (visuospatial modality) with affective information.

The visual and auditory stimuli were adapted to include test-related content (e.g., words such as “exam” or “quiz” and images such as an exam paper or exam class). These stimuli were validated by study participants one week prior to their participation in the study. Specifically, participants were shown a series of test-related words and images and rated their level of anxiety to the stimuli on a scale from 1 (*minimum anxiety*) to 10 (*maximum anxiety*). The 10 stimuli that received the highest anxiety ratings, on average, were subsequently included in the aWMT. The mean anxiety ratings for the images and words included in the training ranged from 5.75 to 8.12 and 6.52 to 8.08, respectively.

Participants heard an unpleasant tone for missed auditory targets and a pleasant tone if the target was identified accurately. For visual targets, a green smiley face appeared following correct identification and a red sad face appeared for missed targets. For each training session, the task commenced at *n* = 1 (i.e., participants had to detect a match between the current stimuli and that presented 1 trial back) and *n* increased by one on the next block when participants detected 60% or more of the targets accurately in a block or reduced by one on the subsequent block if the participants responded to fewer than 20% of the target trials accurately. Each block contained 20 + *n* trials (e.g., when *n* = 1, the block contained 21 trials, when *n* = 2, the block contained 22 trials, etc.). Participants completed 20 blocks per training session.

Participants completed 20 sessions of aWMT on weekdays at the university. While there was no attrition across the course of training, in the case that participants were unable to attend a training session at university, they completed the training session at home on a personal device. Three participants within the aWMT group completed training at home on three days^2^. Training commenced two days after the pre-training assessment. Each training session lasted approximately 30 to 45 min (dependent on the level of *n*-back achieved).

#### Neutral Working Memory

The nWMT was identical to the aWMT except that the stimuli were neutral (e.g., words such as “water”, “hand” and images such as glass, mountain). Mean anxiety ratings for neutral images and words included in the training ranged from 1.20 to 1.87 and 1.10 to 1.28, respectively. The training schedule, timing, and location of the nWMT was also identical to that of the aWMT. While there was also no attrition in the nWMT group, three participants within the nWMT group completed training at home on two days^2^.

### Affective Transfer Measures

The affective transfer measures included in the present study had adequate to excellent internal consistency, which is reported below using Revell’s total omega (*ω_T_*). *ω_T_* has been shown to overcome the limitations and stringent assumptions of Cronbach’s alpha (McNeish, 2018). While there is no universal guide to evaluate *ω_T_*, scores of.50 and higher are considered to reflect acceptable internal consistency (Watkins, 2017).

#### Test Anxiety

The Test Anxiety Inventory (TAI; Spielberger, 1980) was administered to assess TA. The 20-item measure includes an emotionality and worry subscale. Participants are asked to rate how frequently they experience specific symptoms of anxiety before, during, and after examinations on a 4-point scale, ranging from 1 (*almost never*) to 4 (*almost always*). The TAI is commonly used to measure TA (Chapell et al., 2005) and has demonstrated good test-retest reliability (Spielberger, 1980). The Farsi version of the questionnaire has also shown good psychometric properties (Abolghasemi et al., 2004). The TAI demonstrated acceptable internal consistency in the current study (*ω_T_* at pre: 0.72; *ω_T_* at post: 0.88).

#### Emotion Regulation

The Emotion Regulation Questionnaire (ERQ; Gross & John, 2003) was administered to assess participants habitual use of emotion regulation strategies (specifically, cognitive reappraisal and suppression). On the ERQ participants rate ten items on a 7-point Likert scale ranging from 1 (*Strongly disagree*) to 7 (*Strongly agree*). The Farsi version of the questionnaire has demonstrated adequate psychometric properties (Soleimani & Habibi, 2015), and good internal consistency was observed in the current study (*ω_T_* at pre: 0.72; *ω_T_* at post: 0.81).

### Cognitive Transfer Measures

#### Cognitive Control

The GoNogo task (Hester et al., 2004) provided a measure of cognitive control. In this task, cognitive control is required to inhibit prepotent motor responses to infrequent Nogo trials, in the context of response readiness to frequent Go trials (Hester et al., 2004). In the current study, this task was programmed using Super lab-4 software and was completed on a laptop. The task comprised presentation of 100 trials depicting one of seven possible geometric shapes for 500ms each. Participants were required to respond as soon as possible after seeing a triangle by pressing the space bar (70 trials) and refrain from responding to all other shapes (30 trials). Three separate indices were derived from the task: commission errors (keypress for non-targets), omission errors (no keypress for targets) and reaction time on correct trials. In line with Schweizer et al. (2017), we used omission errors as a primary index of cognitive change.

#### Working Memory

The backward version of the digit span task (Wechsler, 1997) was administered as an untrained measure of WM. In this task the experimenter reads a series of random single-digit numbers, beginning with a series of two digits. Participants are asked to repeat the digits in reverse order. After each trial, one digit is added to the series of numbers to reach a maximum of seven digits, or until a series of numbers is repeated incorrectly twice in a row.

### Procedure

Prior to completing the study, informed consent was obtained from all participants. At a pre-training assessment session, participants completed the set of questionnaires as well as the cognitive transfer measures. Before completing the session, participants received an explanation of their assigned training task. Participants then completed their assigned training. The study was single-blind, such that participants were unaware of their assigned training condition, however the experimenter (ZS) was not blind to study condition. The control group did not receive any training. At the post-training assessment session (two days after completion of training), which included all the measures and tasks administered at pre-training, participants were debriefed and compensated with a movie voucher. All computer-based testing and training was done in groups, on a 15-inch laptop and under the supervision of a psychology researcher. This study was approved by the ethics committee of Azarbaijan Shahid Madani University.

### Statistical Analyses

To explore the first *training hypothesis*, that the two training groups were able to train on their respective tasks, a mixed between-within ANOVA was conducted. Time (pre- vs. post-training) was included as the within subject variable and mean level of *n*-back achieved as the dependent variable.

The second *cognitive transfer hypothesis* was investigated with a mixed between-within ANOVA. Time (pre- vs. post-training) was included as the within subject variable and the primary index of cognitive change (omission errors) as the dependent variable. As we did not expect the different forms of training to produce differential effects on cognitive outcomes, we pooled the aWMT and nWMT groups together to form a larger ‘training group’, which was compared to the control group. To investigate the third *affective transfer hypothesis* a mixed between-within MANOVA was conducted, specifying time as the within subject variable and total TA symptoms, emotionality symptoms of TA, worry symptoms of TA, cognitive reappraisal, and suppression as the dependent variables. As we expected aWMT and nWMT to lead to differential effects on affective functioning, the MANOVA compared all three groups (i.e., aWMT group, nWMT group, control group). Next, mediation analyses were conducted to determine whether any affective transfer effects were mediated by changes in cognitive control or changes in emotion regulation, specifically, cognitive reappraisal. In line with Schweizer et al. (2017), we used omission errors as an index of cognitive change, however, the pattern of results did not change when a composite measure of cognitive control (averaged z-transformed difference scores on all cognitive outcome measures) was entered into the analyses. All analyses were conducted with IBM SPSS Statistics 26, with Hayes Process Model 4 used to test mediation. Given the number of univariate comparisons, a Bonferroni-corrected alpha level of 0.01 (0.05/4 [four cognitive and four affective outcomes]) was applied to all analyses.

## Results

### Group Characteristics

For an overview of all group characteristics see Table 1. Average levels of TA symptoms reported in the sample at baseline were *M* = 64.80 (*SD* = 4.73), indicating a high level of TA, when compared to the norms identified in an undergraduate college sample by Spielberger (1980).

### Training Effects

The *training hypothesis* (H1) was only partially supported. The mixed between-within ANOVA revealed a significant time by group interaction (*F* (1, 38) = 15.68, *p* < 0.001, *η_p_^2^* = 0.29), indicating that the change in *n*-back from pre- to post-training varied as a function of training group. Follow-up paired samples t-tests revealed that only the aWMT group demonstrated a significant increase in the mean level of *n*-back achieved from the first day to the last day of training (*M*_Day1_ = 2.35, *M*_Day20_ = 3.90; *t* (19) = −7.34, *p* < 0.001, 95% CI [−1.99, −1.11]). The increase in the nWMT group was non-significant (*M*_Day1_ = 2.90, *M*_Day20_ = 3.05; *t* (19) = −0.53, *p* = 0.603, 95% CI [−0.74, 0.44]).

### Cognitive Transfer Effects

All descriptive statistics for the cognitive measures at pre- and post-training are reported in Table 2. In line with our second *cognitive transfer hypothesis*, comparing change in omission errors from pre- to post-training between the pooled training group and the control group, revealed a significant time by group interaction (Wilks’ Lambda = 0.71, *F* (1, 58) = 23.25, *p* < 0.001, *η_p_^2^* = 0.29). Compared to the control group, the pooled training group demonstrated greater cognitive change (i.e., a greater decrease in omission errors) from pre- to post-training. This pattern of results held for all other cognitive measures. That is, compared to the control group, the training group showed a greater decrease in commission errors and reaction time as well as a greater increase in digits recalled on the backward digit span task from pre- to post-training (Table 2).

Decomposing this significant interaction further, we analysed the effect of time on cognitive change in the training group and control group separately. As expected, the training group showed a significant decrease in omission errors from pre- to post-training (*t* (39) = 8.10, *p* < 0.001, 95% CI [4.01, 6.69]). Conversely, the control group did not show a significant change in omission errors from pre- to post-training (*t* (19) = 0.28, *p* = 0.786, 95% CI [−1.32, 1.72]). This pattern of results held for all other cognitive measures.

To determine whether there were any differential effects on the cognitive transfer measures from pre- to post-training between the two training groups, an additional mixed between-within ANOVA was conducted, comparing the aWMT and nWMT groups. The time by group interaction was not significant according to our Bonferroni-correct alpha level (*F* (1, 38) = 4.56, *p* = 0.039, *η_p_* ^2^ = 0.11), indicating that the two training groups did not show a significant differential decrease in omission errors from pre- to post-training. Similarly, when investigating transfer effects on the secondary cognitive measures (i.e., commission errors, reaction time, and backward digit span), differential effects were not observed between the aWMT group and nWMT group (see Table 2).

### Affective Transfer Effects

All descriptive statistics for the affective measures at pre- and post-training are reported in Table 3. To investigate transfer effects on measures of affective functioning following training, a mixed between-within MANOVA was conducted comparing all three groups. In line with our third *affective transfer hypothesis*, the overall time by group multivariate interaction was significant (Wilks’ Lambda = 0.16, *F* (8, 108) = 20.64, *p* < 0.001, *η_p_^2^* = 0.61). Univariate tests revealed that the significant overall multivariate interaction was being driven by significant time by group interactions on all affective outcome variables (see Table 3). Thus, change in total TA symptoms, emotionality symptoms of TA, worry symptoms of TA, cognitive reappraisal, and suppression from pre- to post-training significantly differed between the three groups.

In order to decompose these significant interactions, additional between-within MANOVAs were conducted comparing the control group to each training group separately and comparing the two training groups to one-another. The time by group interaction was significant when comparing the control group to both the aWMT (Wilks’ Lambda = 0.12, *F* (4, 35) = 62.22, *p* < 0.001, *η_p_^2^* = 0.88) and nWMT (Wilks’ Lambda = 0.33, *F* (4, 35) = 17.88, *p* < 0.001, *η_p_^2^* = 0.67). Univariate tests revealed that the significant overall multivariate interaction was being driven by significant time by group interactions on most affective outcome variables (see Table 3). Thus, compared to the control group, the aWMT group demonstrated a greater decrease in overall TA symptoms, as well as emotionality and worry symptoms of TA, and greater improvements in emotion regulation (i.e., increased cognitive reappraisal and reduced suppression) from pre- to post-training. The nWMT showed the same pattern of results with the exception of suppression.

**Figure 2:**
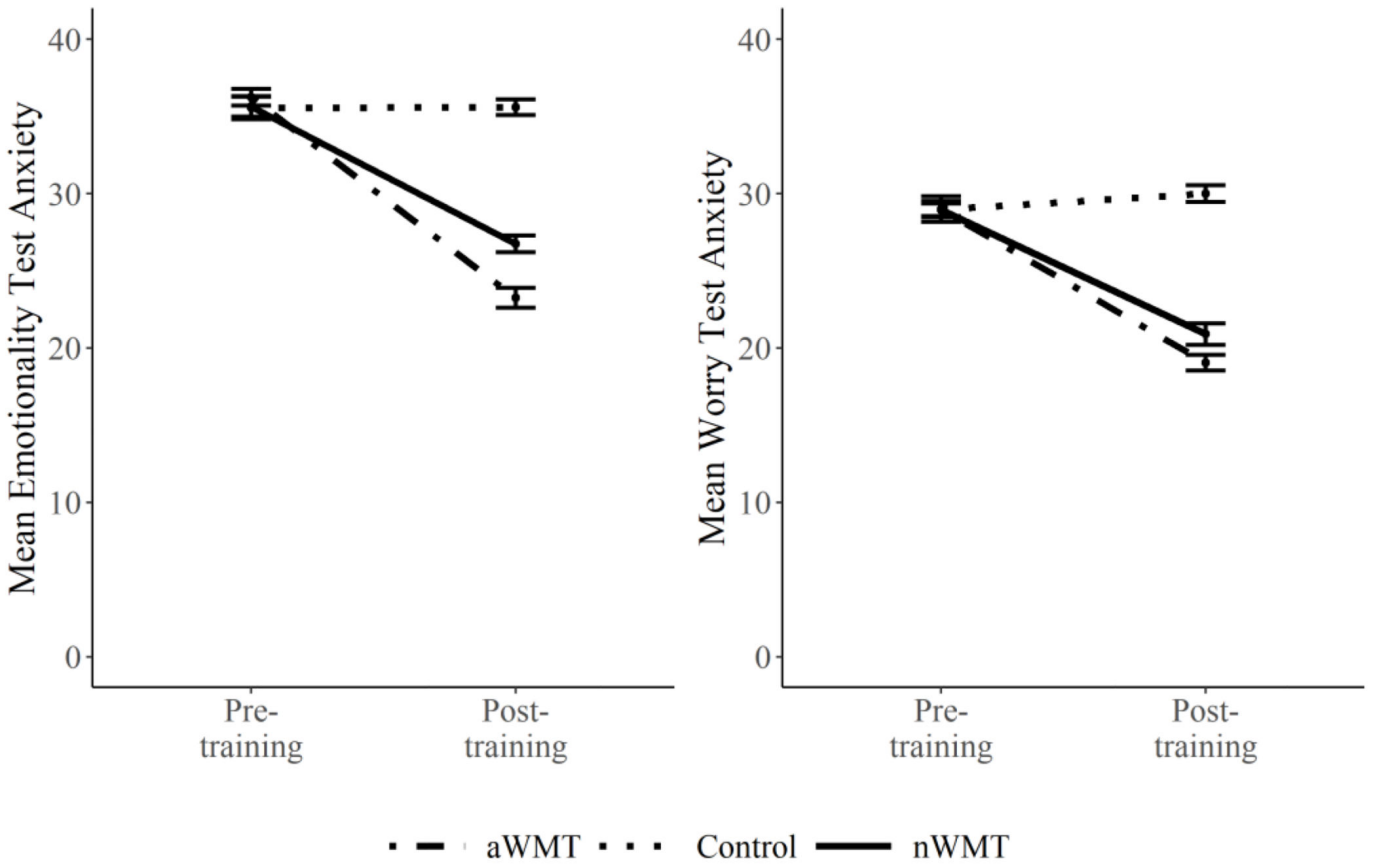
Pre- to Post-Training Changes in Emotionality and Worry Symptoms of TA Across Groups *Note*. aWMT = affective working memory training; nWMT = neutral working memory training.

When comparing the two training groups there was a significant overall time by group multivariate interaction (Wilks’ Lambda = 0.35, *F* (4, 35) = 16.08, *p* < 0.001, *ηp^2^* = 0.65). Univariate tests revealed that this significant overall interaction effect was being driven by significant time by group interactions on emotionality symptoms of TA and cognitive reappraisal, but not the worry symptoms of TA or suppression (see Table 3 and Figure 2).

Decomposing these significant interaction effects further, we analysed the effect of time on affective outcomes in the two training groups and the control group separately. As expected, the aWMT group showed improvement on all affective outcome variables from pre- to post-training (TA – *F* (1, 19) = 267.24, *p* < 0.001, *ηp^2^* = 0.93; TA emotionality – *F* (1, 19) = 203.23,*p* < 0.001, *ηp^2^* = 0.92; TA worry – *F* (1, 19) = 156.14, *p* < 0.001, *ηp^2^* = 0.89; cognitive reappraisal – *F* (1, 19) = 550.37, *p* < 0.001, *ηp^2^* = 0.97; suppression – *F* (1, 19) = 13.07, *p* = 0.002, *ηp^2^* = 0.41). Contrary to hypotheses, the nWMT group also showed improvement on the majority of affective outcome variables from pre- to post-training (TA – *F* (1, 19) = 118.80, *p* < 0.001, *ηp^2^* = 0.86; TA emotionality – *F* (1, 19) = 81.84, *p* < 0.001, *ηp^2^* = 0.81; TA worry – *F* (1, 19) = 82.37, *p* < 0.001, *ηp^2^* = 0.81; cognitive reappraisal – *F* (1, 19) = 76.77, *p* < 0.001, *ηp^2^* = 0.80). However, the nWMT did not demonstrate reduced suppression from pre- to post-training (*F* (1, 19) = 0.00, *p* = 1.00, *ηp^2^* = 0.00). Unexpectedly, we observed a significant overall effect of time on affective outcome variables in the control group (Wilks’ Lambda = 0.43, *F* (4, 16) = 5.39, *p* = 0.006, *ηp^2^* = 0.57). Univariate tests revealed that this significant multivariate effect of time was being driven by a significant increase in cognitive reappraisal across time (*F* (1, 19) = 16.64, *p* < 0.001, *ηp^2^* = 0.47; all other univariate *F*’s < 2.04, *p*’s > 0.169).

Finally, we explored whether these affective gains were mediated by changes in cognitive control or emotion regulation from pre- to post-training. However, the effect of training group on change in affective outcomes was not mediated by pre- to post-training improvements in cognitive control. Standardised indirect effects were: TA symptoms: *B* = −0.06, 95% CI [−0.19, 0.07]; emotionality symptoms of TA: *B* = −0.08, 95% CI [−0.20, 0.04]; worry symptoms of TA: *B* = −0.03, 95% CI [−0.17, 0.11]; cognitive reappraisal: *B* = 0.06, 95% CI [−0.06, 0.19]; suppression: *B* = 0.03, 95% CI [−0.13, 0.19]). Similarly, the effect of training group on change in TA symptoms was not mediated by pre- to post-training changes in cognitive reappraisal. Standardised indirect effects were: TA symptoms: *B* = 0.08, 95% CI [−0.11, 0.30]; emotionality symptoms of TA: *B* = 0.02, 95% CI [−0.17, 0.24]; worry symptoms of TA: *B* = 0.13, 95% CI [−0.08, 0.36].

## Discussion

The current study aimed to explore the impact of aWMT on cognitive and affective functioning in university students with TA. Significant improvements from pre- to post-training on the training task were observed only in the aWMT, but not the nWMT group. Despite the lack of training related improvement in the nWMT, compared to the no training control group, both aWMT and nWMT led to improved cognitive and affective functioning. Affective transfer effects, however, were greater in the aWMT group compared to the nWMT group. Specifically, the aWMT group demonstrated a greater reduction in overall TA symptoms and emotionality symptoms of TA and a greater increase in cognitive reappraisal from pre- to post-training, compared to the nWMT group. The two forms of training did not have a differential impact on worry symptoms of TA or on suppression. When investigating the impact of time on affective outcomes in the two training groups separately, we observed decreased TA symptoms, including both emotionality and worry symptoms of TA, and increased cognitive reappraisal in both groups from pre- to post-training. However, only the aWMT group showed decreased suppression from pre- to post-training.

The results of the study suggest that WM training, in particular, aWMT, may be an effective means of reducing symptoms of TA, as well as associated cognitive and affective impairments. To combat emotionally-laden thoughts and feelings in everyday life, both healthy populations as well as individuals with emotional disorders, such as TA, require affective WM (Shi et al., 2014). However, the literature suggests that affective WM capacity is impaired in individuals with mental health difficulties (Schweizer et al., 2019), including in individuals with TA (Shi et al., 2014). The finding that affective WM can be trained in individuals with TA, then, is particularly promising. Also encouraging, was the finding that aWMT produced both cognitive and affective transfer effects, specifically, improved working memory, cognitive control, TA symptoms, and emotion regulation. This is consistent with previous research, showing improved cognitive control over affective information and an increase in adaptive emotion regulation in psychologically healthy individuals following aWMT (Schweizer et al., 2011, 2013) as well as a study finding improved emotion regulation and PTSD symptoms in adolescents following aWMT (Schweizer et al., 2017).

aWMT arguably offered participants the opportunity to practice the mental manipulation of *emotional* stimuli, perhaps making it easier for these participants to mentally engage or disengage from or shift attention towards or away from negative emotional material (such as negative test-related thoughts or feelings), and consequently resulting in overall better mental control over such emotional stimuli (du Toit et al., 2020). Moreover, affective WM shares the same frontoparietal neural circuitry as emotion regulation capacity; circuitry which has demonstrated improved efficiency following aWMT (Pan et al., 2020; Schweizer et al., 2013). Given that TA can have a detrimental impact on an individual’s academic performance, implementing such simple and cost-effective cognitive training procedures as aWMT in educational settings may enable students to overcome symptoms of TA and perform to their highest potential in evaluative situations.

The finding that participants who received nWMT also demonstrated improved TA symptoms and emotion regulation following training was contrary to our hypotheses, though nonetheless a promising finding and in line with Motter and colleagues′ (2016) review of the literature on neutral cognitive training. Given that we did not include an active control group, we cannot rule out the possibility that the beneficial effects we observed following training were an impact of placebo effects associated with study participation, such as regular interpersonal interaction with the research team or participants expecting to improve as a result of training. Alternatively, perhaps the training sessions were somewhat akin to exposure, such that participants were repeatedly exposed to test-like settings, where their performance on the training task was being evaluated by a researcher. If there were indeed any effects of exposure, this effect would likely have been potentiated in the aWMT group. This is because one of the study’s strengths was that the aWMT included TA-related stimuli that were rated as most distressing by the participants prior to the training. In order to rule out these alternative explanations, future research comparing aWMT and nWMT should also include a placebo task, including the same affective stimuli as the aWMT, but excluding the WM training component.

Further contrary to our hypotheses, the impact of training on affective outcome variables was not mediated by changes in cognitive control or emotion regulation. These findings are in contrast with previous studies showing training gains to be mediated by improved affective and cognitive control (Schweizer et al., 2013, 2017). We may have failed to observe this expected relationship as we did not include a measure of *affective* control. It may be that the improved affective functioning we observed following training, particularly in the aWMT group, was a result of improved affective control, rather than cognitive control *per se*. Future studies would benefit from including a measure of affective control, such as the affective GoNogo task or affective Stroop task, in order to assess this hypothesis.

The failure of the nWMT group to show training-related improvement on the training task was surprising. Although the two training groups did not significantly differ in mean *n*-back achieved at Day 1 of training, the mean for the nWMT group was nonetheless slightly higher, suggesting that perhaps there was less room for improvement in this group. The mean *n*-back achieved at pre-training for the nWMT group also exceeded that observed in previous *n*-back studies (e.g., du Toit et al., 2020; Sari et al., 2016; Schweizer et al., 2013). Moreover, the range of *n*-back scores at pre-training for the nWMT group was greater (range: 1 – 6) than the range of scores for the aWMT group (range: 1 – 4). It is possible that the nWMT group included participants who performed at ceiling on this task at pre-training, which may account for the non-significant training improvement in this group.

While the findings from the current study are promising, they must be interpreted within the context of the limitations of the study. First, while the inclusion of both an aWMT group and nWMT group was beneficial, the study was limited by the inclusion of a passive rather than active control group (Redick, 2019). Future studies would benefit from the inclusion of a placebo-training task, which is similar in duration and structure to the active training task, to allow for greater confidence that any significant results observed are not merely an impact of participants expecting to benefit from training. However, meta-analytic evidence suggests that the type of control group (active vs. passive) does not moderate the impact of training on transfer effects (Au et al., 2015; Soveri et al., 2017). Second, allowing participants to complete training at home on personal devices represents a further limitation to the study. Not only may this be seen as avoidance on the participants’ behalf, but it also reduces control over training settings. However, the controlled training setting also limits applicability to more naturalistic environments. Future research in which all training is conducted in the same setting will be important to replicate effects, followed by more naturalistic training studies, to determine applicability to real-life contexts. Third, we did not collect arousal ratings pre- and post-training. These ratings would help to determine whether the two training settings, which are somewhat similar to test settings, differentially served as an anxiety induction. If they did indeed induce anxiety, training may by extension potentially have served as exposure, which in turn could account for the beneficial effects of the training. A fourth limitation was the single-blind nature of the study, with the same experimenter, who was aware of training condition, conducting the clinical interviews and the pre- and post-assessments. Fifth, the current study does not include task reliability for the cognitive transfer task as the data was not available at the review stage. However, the GoNogo task has been shown to have good reliability when at least 50-80 trials are included (Williams & Kaufmann, 2012). Here we included 100 trials, increasing the confidence in the psychometric adequacy of the task. Finally, the current study was limited by a small sample size. Consequently, the results we obtained are likely to represent an overestimation of the effect in the general population (Redick, 2019; Schweizer et al., 2017). Future studies with larger sample sizes will be important to determine the replicability of the effects reported. Longer-term follow-ups are also needed to determine whether any beneficial effects of aWMT or nWMT persist beyond post-training assessments. Such studies would also allow for investigation of whether transfer effects following WM training extend beyond cognitive and affective functioning to more practical outcomes, such as improved academic performance.

The current study adds to the existing literature by providing evidence that both aWMT and nWMT can lead to improved cognitive and affective functioning in university students with TA, though the impact on affective functioning appears to be greater following aWMT. Given the ease and cost-effectiveness of administering cognitive training, our findings highlight the potential of aWMT as a promising avenue for improving symptoms of TA, which may allow students to perform to their full academic potential in evaluative situations.

## Figures and Tables

**Table 1:** Demographic and Baseline Clinical and Cognitive Characteristics

	aWMT	nWMT	Control
	*n* = 20	*n* = 20	*n* = 20
Demographic			
Age in years, *M* (*SD*)	20.50 (1.15)	20.40 (1.05)	20.60 (1.10)
Academic year, 1:2:3:4	5:5:5:5	4:8:4:4	4:5:4:7
Female, *n*	10	10	10
SES, High:Middle:Low	0:20:0	0:20:0	0:20:0
Clinical			
Total test anxiety, *M* (*SD*)	65.25 (3.68)	64.60 (3.78)	64.55 (6.44)
Cognitive reappraisal, *M* (*SD*)	18.00 (2.34)	16.70 (4.59)	18.20 (3.05)
Suppression, *M* (*SD*)	11.80 (1.70)	12.20 (3.14)	12.85 (3.67)
Cognitive			
Commission errors, *M* (*SD*)	10.80 (2.78)	11.00 (2.87)	10.80 (2.76)
Omission errors, *M* (*SD*)	12.80 (3.11)	12.45 (3.49)	12.40 (2.66)
Reaction time, *M* (*SD*)	293.85 (52.51)	324.00 (44.96)	294.60 (47.29)
Digit backward, *M* (*SD*)	6.40 (1.19)	6.10 (1.02)	6.30 (1.03)

*Note*. aWMT = affective working memory training; nWMT = neutral working memory training; SES = socioeconomic status based on family’s annual household income.

**Table 2:** Descriptive Statistics for Cognitive Outcome Variables and Results from Between by Within MANOVAs Comparing Change in Cognitive Functioning from Pre- to Post-Training Between Groups

	aWMT		nWMT		Control		Pooled training group vs. Control	aWMT vs. nWMT
	Pre-training	Post-training	Pre-training	Post-training	Pre-training	Post-training		
Commission errors, *M* (*SD*)	10.80 (2.78)	8.60 (2.60)	11.00 (2.87)	8.70 (2.68)	10.80 (2.76)	11.85 (1.90)	*F* (1, 58) = 10.32, *p* = 0.002, *η_p_^2^* = 0.15	*F* (1, 38) = 0.01, *p* = 0.938, *η_p_^2^* = 0.00
Omission errors, *M* (*SD*)	12.80 (3.11)	6.10 (2.43)	12.45 (3.49)	8.45 (2.74)	12.40 (2.66)	12.20 (2.53)	*F* (1, 58) = 23.25, *p* < 0.001, *η_p_^2^* = 0.29	*F* (1, 38) = 4.56, *p* = 0.039, *η_p_^2^* = 0.11
Reaction time, *M* (*SD*)	293.85 (52.51)	172.60 (39.59)	324.00 (44.96)	201.45 (39.23)	294.60 (47.29)	304.25 (49.00)	*F* (1, 58) = 47.34, *p* < 0.001, *η_p_^2^* = 0.45	*F* (1, 38) = 0.00, *p* = 0.954, *η_p_^2^* = 0.00
Digit backward, *M* (*SD*)	6.40 (1.19)	9.00 (1.45)	6.10 (1.02)	8.15 (2.01)	6.30 (1.03)	5.65 (1.42)	*F* (1, 58) = 30.11, *p* < 0.001, *η_p_^2^* = 0.34	*F* (1, 38) = 0.74, *p* = 0.396, *η_p_^2^* = 0.02

*Note*. aWMT = affective working memory training; nWMT = neutral working memory training.

**Table 3:** Descriptive Statistics for Affective Outcome Variables and Results from Between by Within MANOVAs Comparing Change in Affective Functioning from Pre- to Post-Training Between Group

	aWMT		nWMT		Control		aWMT vs nWMT vs Control	aWMT vs Control	nWMT vs Control	aWMT vs nWMT
	Pre-training	Post-training	Pre-training	Post-training	Pre-training	Post-training				
Total test anxiety, *M* (*SD*)	65.25 (3.68)	42.30 (3.92)	64.60 (3.78)	47.65 (4.56)	64.55 (6.44)	65.60 (3.72)	*F* (2, 57) = 63.36, *p* < 0.001, *η_p_^2^* = 0.69	*F* (1, 38) = 115.94,*p* < 0.001, *η_p_^2^* = 0.75	*F* (1, 38) = 59.83, *p* < 0.001, *η_p_^2^* = 0.61	*F* (1, 38) = 8.20, *p* = 0.007, *η_p_^2^* = 0.18
Test anxiety – emotionality, *M* (*SD*)	36.25 (2.45)	23.25 (2.92)	35.65 (2.98)	26.75 (2.43)	35.55 (3.33)	35.60 (2.26)	*F* (2, 57) = 48.97, *p* < 0.001, *η_p_^2^* = 0.63	*F* (1, 38) = 96.74, *p* < 0.001, *η_p_^2^* = 0.72	*F* (1, 38) = 42.23, *p* < 0.001, *η_p_^2^* = 0.53	*F* (1, 38) = 9.34, *p* = 0.004, *η_p_^2^* = 0.20
Test anxiety – worry, *M* (*SD*)	29.00 (2.32)	19.05 (2.33)	28.95 (1.79)	20.90 (3.09)	29.00 (3.67)	30.00 (2.41)	*F* (2, 57) = 41.44, *p* < 0.001, *η_p_^2^* = 0.59	*F* (1, 38) = 70.87, *p* < 0.001, *η_p_^2^* = 0.65	*F* (1, 38) = 44.40, *p* < 0.001, *η_p_^2^* = 0.54	*F* (1, 38) = 2.54, *p* = 0.119, *η_p_^2^* = 0.06
Cognitive reappraisal, *M* (*SD*)	18.00 (2.34)	34.20 (2.53)	16.70 (4.59)	27.25 (3.35)	18.20 (3.05)	22.05 (3.07)	*F* (2, 57) = 40.70, *p* < 0.001, *η_p_^2^* = 0.59	*F* (1, 38) = 111.51, *p* < 0.001, *η_p_^2^* = 0.75	*F* (1, 38) = 19.18, *p* < 0.001, *η_p_^2^* = 0.34)	*F* (1, 38) = 16.57, *p* < 0.001, *η_p_^2^* = 0.30
Suppression, *M* (*SD*)	11.80 (1.70)	9.45 (1.96)	12.20 (3.14)	12.20 (1.58)	12.85 (3.67)	14.30 (2.87)	*F* (2, 57) = 5.46, *p* = 0.007, *η_p_^2^* = 0.16)	*F* (1, 38) = 9.95, *p* = 0.003, *η_p_^2^* = 0.21	*F* (1, 38) = 1.32, *p* = 0.258, *η_p_^2^* = 0.03	*F* (1, 38) = 5.57, *p* = 0.023, *η_p_^2^* = 0.13

*Note*. aWMT = affective working memory training; nWMT = neutral working memory training.

## Footnotes

1 Based on an effect size of *d* =.6-1.2 a total sample size of 30 is recommended. However, we doubled the sample to account for the likely overestimation of effect sizes from the previous studies that included ≥ *n* = 17 per group, as well as potential attrition, estimated at 20%.

2 The pattern of results for all hypotheses stayed the same when controlling for the effect of training at home.
